# Early life adversity blunts the subjective and physiological relaxation response in healthy adults

**DOI:** 10.1038/s41598-024-78340-3

**Published:** 2024-11-14

**Authors:** Raphaela J. Gaertner, Manuel Burkart, Louisa Richter, Pius Schnell, Matthias Finkhäuser, Elea S. C. Klink, Bernadette F. Denk, Maria Meier, Ulrike U. Bentele, Stella Wienhold, Katharina E. Kossmann, Jens C. Pruessner

**Affiliations:** 1 Division of Neuropsychology, Department of Psychology, University of Konstanz, Fach 905, Universitaetsstrasse 10, 78464 Constance, Germany; 2 Centre for the Advanced Study of Collective Behaviour, University of Konstanz, Constance, Germany; 3 Child and Adolescent Psychiatric Research Department (UPKKJ), University Psychiatric Clinics (UPK), University of Basel, Basel, Switzerland

**Keywords:** Early life adversity, Relaxation, Paced breathing, Virtual nature, Heart rate variability, Risk factors, Psychology

## Abstract

While Early Live Adversity (ELA) is a known risk factor for mental and physical diseases, the investigation into the mechanisms behind this connection is ongoing. In the present study, we investigated whether ELA blunts the relaxation response in healthy adults. Using a within-subjects design, we employed a paced breathing exercise (four seconds inhale, six seconds exhale) and a 360° nature video as relaxation interventions while measuring physiological relaxation using heart rate variability and subjective relaxation using the Relaxation State Questionnaire. A total of 103 participants (63.11% female; age_mean_ = 22.73 ± 3.43 years) completed the Parental Bonding Instrument and the Childhood Trauma Questionnaire to assess ELA retrospectively. For subjective relaxation, a blunted relaxation reaction was associated with lower scores of paternal care and higher scores of paternal overprotection, physical abuse, physical neglect, and emotional abuse. For heart rate variability emotional abuse in interaction with nicotine consumption was related to a blunted relaxation response. This indicates that experiencing ELA negatively affects the relaxation capability in a healthy sample and emphasizes the importance of assessing relaxation at a physiological and subjective level.

## Introduction

While there is no universal definition for early life adversity (ELA), the term describes stressful events, such as poverty, abuse, bullying, or absent parents, that endanger a child’s mental and physical well-being and often require adaption of behavioral and neurophysiological systems^1,2^. These experiences have been shown to impact not only in childhood but also throughout the lifespan, increasing the risk of developing psychological and physiological disorders^1,3^. However, despite numerous explanatory approaches^2,4^ the underlying mechanisms behind ELA as a risk factor for diseases are unclear.

Previous studies found changes in the autonomic nervous system (ANS) linked with ELA^2^, for example, a decrease in resting state heart rate variability (HRV)^2,5,6^. This implies a distortion of the parasympathetic nervous system (PNS) and, as HRV generally serves as a marker of ANS adaptability^7,8^, could also imply a reduced adaptability to changing environmental demands. Adaptability is an integral part of physical and mental health^7–9^, with decreased resting-state HRV linked to ELA and various mental and physiological diseases^7,10,11^. While numerous studies have found a distortion of the affective and physiological reaction to stress related to ELA^7–13^, there is a considerable lack of studies focusing on the effect on relaxation. A reduced baseline HRV could indicate a blunting in the relaxation response, since relaxation can be seen as an adaptation to safe situations. At the time of writing, no study has investigated possible changes in relaxation reactivity related to ELA.

Relaxation has been linked to an increase in well-being and health, therefore representing an important contribution to a healthy life^14^. There are vast interindividual differences when looking in the effectiveness of relaxation interventions^15^, indicating that multiple factors influence a person’s ability to relax. While this field of research is growing, initial studies report a negative effect of depression^16^ and a positive effect of trait mindfulness^17^ on the relaxation response. Further studies are needed to investigate possible influencing factors using different relaxation methods, especially since there are many different relaxation interventions, such as massages^18^, breathing exercises^19^, or forest bathing^20^. In the current study, we chose a 360° virtual nature video and a breathing exercise because of their easy implementation and different mechanisms behind these interventions. Nature stimuli have been shown to increase subjective and physical relaxation^20,21^, either as real-life stimuli^22^ or as virtual stimuli^23^. The Psychoevolutionary Theory^24^ postulates that humans had an evolutionary advantage linked to their capability to recover quickly in a safe and nonthreatening natural environment, linking nature to a positive emotional reaction^21^. Complementary, breathing exercises are also effective in inducing subjective relaxation^19^, for example, breathing steadily with a slow rhythm (six breaths per minute^25^). Breathing rhythm and heart activity are closely linked via the vagus nerve, therefore a slow breathing rhythm can trigger the physiological relaxation response^26^.

Relaxation can be measured at both subjective and physiological levels. While definitions of relaxation vary, subjective relaxation commonly includes a calm state of mind, specific affective states (e.g., calmness and joy), and a perceived reduction in mental and physiological arousal^27,28^. At the physiological level, reduced arousal is associated with a change in ANS activity, usually an increase in PNS activity and a simultaneous decrease in sympathetic nervous system (SNS) activity. It should be noted that an increase in PNS activity does not necessarily lead to a decrease in SNS activity^29^. A noninvasive and valid way to measure PNS activity is the assessment of vagally mediated HRV, describing the beat-to-beat fluctuation of heart rate^7,30^. There are many factors affecting HRV (e.g., age, sex, health, and breathing rhythm^7,31^) with should be taken into account.

The present study aimed to investigate a possible distortion of the subjective and physiological relaxation responses in healthy participants by ELA using two relaxation interventions. The study was preregistered at OSF before data analysis (https://osf.io/jsrze). We defined physiological relaxation as an increase in parasympathetic activity, as measured by HRV. We chose the root mean square of successive differences (RMSSD) as a marker of HRV, since it adequately represents the increase in PNS activity associated with relaxation independent of breathing cycle^30^. We also assessed subjective relaxation through the Relaxation State Questionnaire (RSQ^28^) since subjective and physiological markers often diverge^32,33^. From the vast range of available ELA measures^34^, we chose to assess two retrospective questionnaires, the German translation of the Parental Bonding Inventory (PBI^35^)41)and the German translation of the Childhood Trauma Questionnaire (CTQ^36^).

We hypothesized an increase in physiological (RMSSD) and subjective (RSQ) relaxation markers in reaction to both relaxation interventions. Specifically, we expected that the breathing exercise would solely increase RMSSD during the intervention, whereas the nature video would not only induce relaxation during the video but also maintain a relaxing effect after the VR session. For the influence of ELA, we predicted that a blunted relaxation response in both relaxation interventions would be associated with higher scores in both forms of ELA.

## Results

### Subjective relaxation (RSQ)

**Fig. 1:**
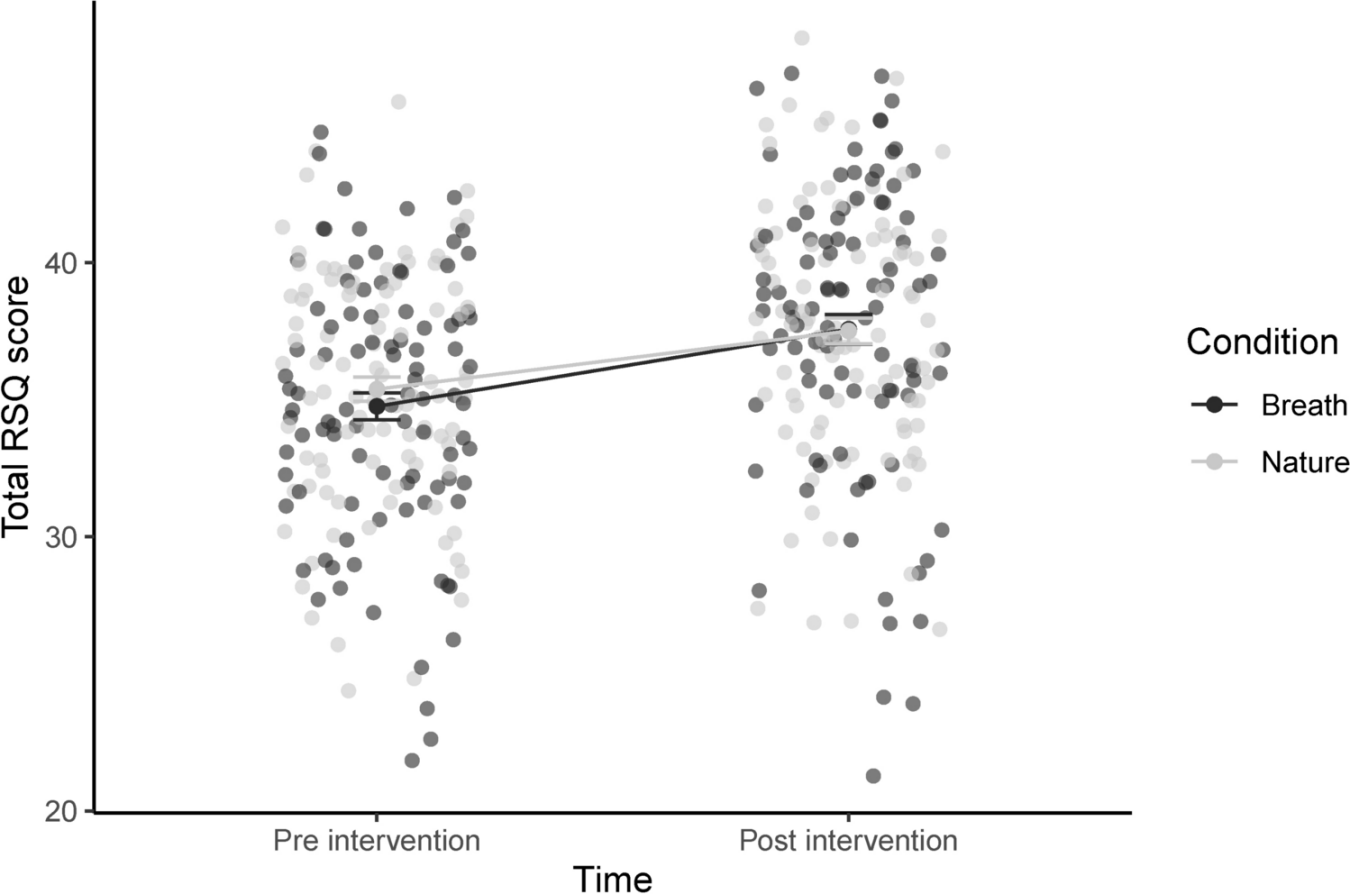
Changes in total RSQ scores for both experimental interventions (nature and breath) with individual data points. Error bars indicate the standard error.

We found a significant main effect of time (*p* < 0.001; *η*^2^ = 0.06), indicating a significant increase in RSQ in reaction to both relaxation interventions (see Fig. 1). The increase in RSQ scores (main effect of time) remained significant after controlling for all covariates by conducting ANCOVAs.

Maternal care and overprotection had no significant effect on the increase in RSQ induced by both relaxation interventions. The main effects of paternal care (*p* < 0.01; *η*^2^ = 0.03) and paternal overprotection (*p* = 0.01; *η*^2^ = 0.02) each reached statistical significance in addition to the increase in RSQ scores (main effect of time). Post-hoc tests revealed that overall lower subjective relaxation scores before and after the interventions were associated with lower paternal care (*r* =  − 0.24) and higher paternal overprotection (*r* = 0.21).

Of the CTQ subscales sexual abuse and emotional neglect had no significant effect. In addition to the increase in RSQ scores (main effect of time), we found a significant main effect of emotional abuse (*p* < 0.05; *η*^2^ = 0.02), with post hoc tests showing that higher scores of emotional abuse were associated with lower RSQ scores before and after the interventions (*r* =  − 0.14). Physical abuse (*p* < 0.01; *η*^2^ = 0.01) and physical neglect (*p* < 0.05; *η*^2^ = 0.009) both showed a significant interaction with the changes in RSQ induced by the interventions. As post-hoc test, we calculated the area under the curve with respect to increase (AUCi^37^) as a measure of change in RSQ scores incuced by the interventions. We found smaller changes in RSQ to be associated with higher scores in physical abuse and physical neglect. See Table 1 for an overview of the parameters of all ANOVAs that indicated significant effects.

**Table 1:** Parameters of all ANOVAs that indicated significant effects.

Effect	DFn	DFd	*F*	*p*
ANOVA effect of interventions				
Condition	1	190	0.25	0.62
Time	1	190	40.26	** < 0.001**
Time × condition	1	190	0.78	0.38
ANOVA paternal care				
Condition	1	188	0.26	0.61
Paternal care	1	188	9.67	**0.03**
Time	1	188	41.03	** < 0.001**
Condition × paternal care	1	188	0.001	0.97
Condition × time	1	188	0.80	0.37
Paternal care × time	1	188	3.05	0.08
Condition × paternal care × time	1	188	2.59	0.11
ANOVA paternal overprotection				
Condition	1	188	0.26	0.61
Paternal overprotection	1	188	6.68	**0.01**
Time	1	188	40.17	** < 0.001**
Condition × paternal overprotection	1	188	2.35	0.13
Condition × time	1	188	78	0.22
Paternal overprotection × time	1	188	1.50	0.22
Condition × paternal overprotection × time	1	188	0.09	0.77
ANOVA emotional abuse				
Condition	1	188	0.25	0.61
Emotional abuse	1	188	5.63	**0.02**
Time	1	188	40.51	** < 0.001**
Condition × emotional abuse	1	188	0.02	0.88
Condition × time	1	188	0.79	0.38
Emotional abuse × time	1	188	2.72	0.10
Condition × emotional abuse × time	1	188	2.71	0.10
ANOVA physical abuse				
Condition	1	188	0.24	0.62
Physical abuse	1	188	0.03	0.85
Time	1	188	41.70	** < 0.001**
Condition × physical abuse	1	188	0.002	0.96
Condition × time	1	188	0.81	0.37
Physical abuse × time	1	188	8.66	**0.003**
Condition × physical abuse × time	1	188	0.17	0.68
ANOVA physical neglect				
Condition	1	188	0.25	0.62
Physical neglect	1	188	1.54	0.22
Time	1	188	41.08	** < 0.001**
Condition × physical neglect	1	188	1.89	0.17
Condition × time	1	188	0.80	0.37
Physical neglect × time	1	188	5.52	0.**01**
Condition × physical neglect × time	1	188	0.38	0.54

Time describes the changes in RSQ values induced by the interventions; condition describes the relaxation interventions breath and nature; paternal neglect and paternal overprotection derived from the PBI; emotional abuse, physical abuse, and physical neglect derived from the CTQ.

### Physiological relaxation (RMSSD)

**Fig. 2:**
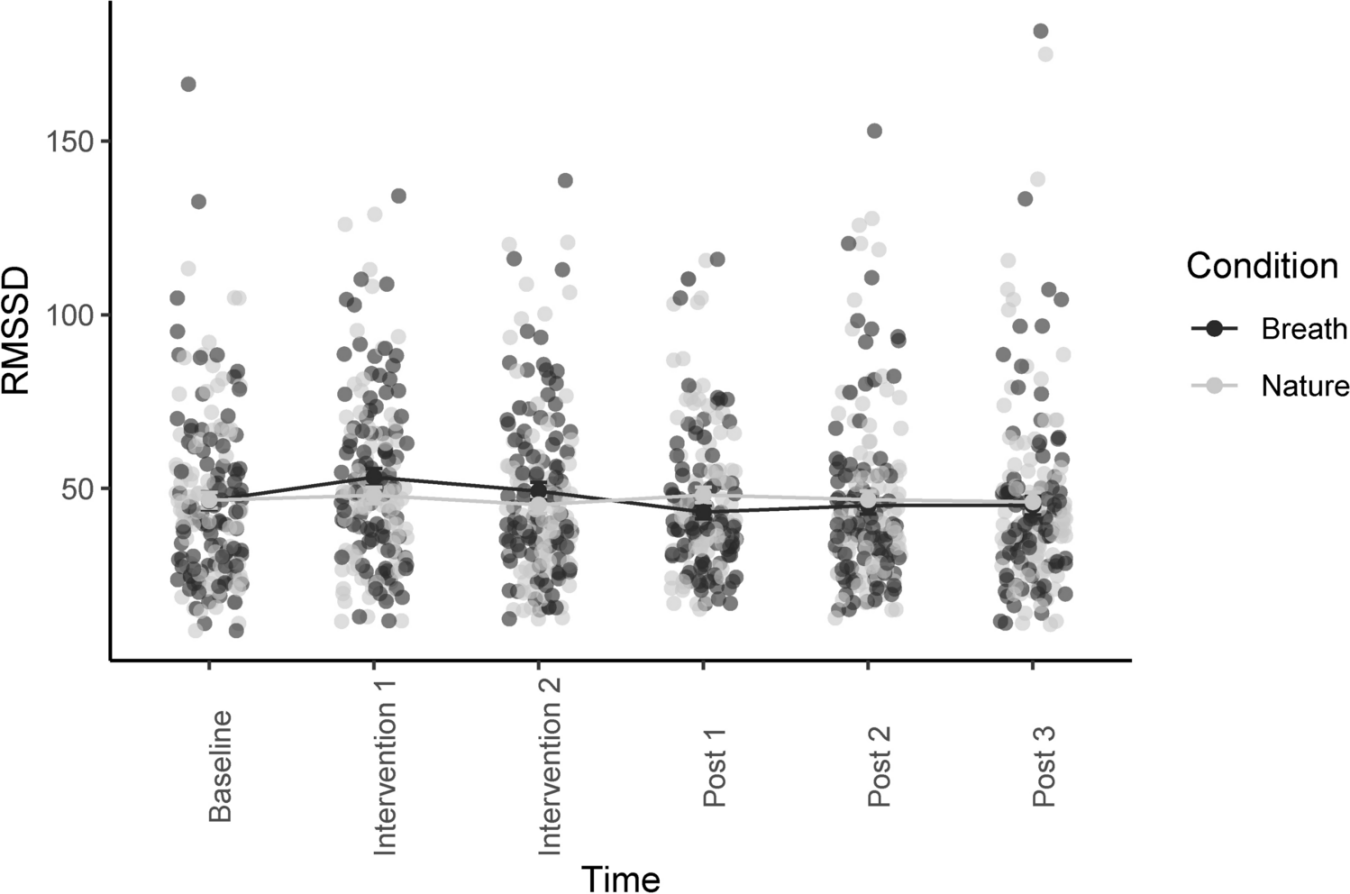
Changes in RMSSD over time in the two relaxation conditions (nature and breath) with individual data points. Error bars depict the standard error.

Figure 2 depicts a graphical representation of the change in RMSSD over time. The basic model included a random intercept and a random cubic slope. The Intra-class Correlation Coefficient was 70.95%. Adding a main effect of condition significantly improved the model fit, as did the interaction between changes in RMSSD and condition. This indicates that only the breath condition significantly increased RMSSD during the breathing exercise. We added each subscale of PBI and CTQ separately to the model to test if they significantly increased the model fit, which was only the case for the main effect of emotional abuse. Higher emotional abuse scores were associated with blunted physiological relaxation. Of the covariates only nicotine consumption had a significant influence on the relationship between the predictors and RMSSD as outcome variable. Interestingly, adding the effect of nicotine consumption to the model affected the main effect of emotional abuse, attenuating its influence so that it did not reach significance. Therefore, we added the interaction of emotional abuse and nicotine consumption, which increased the overall model fit. In this new model, only the interaction of emotional abuse and nicotine consumption reached statistical significance, while both main effects were not statistically significant. This indicates that nicotine consumption seems to mediate the effect of emotional abuse on the physiological relaxation reaction. For detailed parameters of the final model see Table 2.

**Table 2:** Parameters of the final model for RMSSD.

Predictors	Value		
	Estimates	CI	*p*
(Intercept)	43.76	35.10–52.42	** < 0.001**
Time	− 1.09	− 1.75– − 0.43	**0.001**
Condition [nature]	− 2.95	− 5.29– − 0.60	**0.014**
Emotional abuse	0.63	− 0.35–1.61	0.204
Nicotine	− 5.23	− 13.31–2.85	0.203
Time : condition [nature]	1.00	0.23–1.78	**0.011**
Emotional abuse :nicotine	0.67	0.04–1.30	**0.039**
N_code_	103		
Observations	1236		

Emotional abuse is the sum score of the CTQ subscale “emotional abuse”; nicotine consumption was assessed as cigarettes consumed per day.

## Discussion

The aim of this research was to investigate whether experiencing ELA leads to a distortion of the subjective and physiological relaxation response in reaction to a paced breathing exercise and a nature video in healthy participants. We found that both interventions successfully increased subjective relaxation. Concerning the influence of ELA, we found the subjective relaxation reaction to be blunted in association with lower paternal care, higher paternal overprotection, and higher scores on emotional abuse, physical abuse, and physical neglect. On a physiological level, we found an increase in RMSSD evoked only by the paced breathing intervention during the intervention. Higher emotional abuse scores in interaction with nicotine consumption were associated with a blunted physiological relaxation response.

Both interventions being similarly effective in increasing subjective relaxation fits with previous studies reporting changes in affect associated with relaxation in response to nature stimuli^21^ and paced breathing interventions^19^. Only the breathing exercise leading to an increase in physiological relaxation partly supports our hypotheses, since we expected an increase in subjective and physiological relaxation in response to both interventions. The increase in RMSSD induced by a breathing rhythm of six breaths per minute replicates findings from previous research^25,37–39^. The nature video being not effective in increasing RMSSD fits with the ambiguous picture in the current literature^33^, with some studies finding an effect^40^ while others did not^41^. Several factors may explain why our intervention did not significantly increase RMSSD. Since the quality of the nature video was 4K and lacked tactile or olfactory impressions, the evoked experience may not felt real enough to elicit a physiological relaxation response. In this sample, the vast majority had rarely or never used VR, therefore using VR could have been be experienced as stressful or exciting, with the possibility of masking potential relaxing effects of the nature video. Furthermore, ceiling effects are an issue when inducing relaxation^42^, possibly masking the relaxing effects of the nature video in our study, since we did not stress participants prior to the relaxation interventions.

While for subjective relaxation various forms of ELA were found to be influential, only the influence of emotional abuse in interaction with smoking reached statistical significance for physiological relaxation. Both the PBI and CTQ questionnaires were used to evaluate self-reported ELA, which may elucidate the greater impact on subjective relaxation compared to physiological relaxation. Perhaps the subjective feeling of relaxation is subconsciously linked to the overall subjective evaluation of life experiences. Similar to the negativity bias^43^ this could associate perceiving events as more negative with a general focus on negative experiences. Difficulties in experiencing subjective relaxation might in turn reduce the subjective relaxation capability.

While there was no main effect of any of the ELA scores, the interaction of emotional abuse and nicotine consumption blunted the physiological relaxation reaction. This finding is consistent with previous studies that have found ELA to be associated with an increase in health risk behaviors such as nicotine consumption^44^, as well as an association between reduced resting state HRV, smoking^45^ and ELA^2^. In addition to these effects on baseline HRV the current findings indicate that there seems to be a reduced reactivity to relaxation interventions associated with smoking and ELA, even in a healthy sample.

The link between ELA, lower HRV, and psychopathology is still unclear, with multiple pathways forming plausible theoretical explanations for associations. First, ELA could lead to maladaptive emotion regulation strategies, increasing the risk of psychopathology which in turn leads to a lower HRV^4^. Second, ELA could directly lead to dysregulation in the ANS—indicated by lower HRV—which then affects emotion regulation and leads to psychopathology. Third, ELA could affect brain development which then affects emotion regulation and HRV independently^2^. In turn, higher HRV is associated with higher emotion regulation capabilities and adequately rating safe environments as such^8^. While the mechanisms behind the association between ELA, lower HRV, and psychopathology are still unclear, there is considerable evidence linking ELA to physical and mental diseases^32,44,46,47^.

A distortion of the relaxation response in addition to the distorted baseline HRV could add another factor to the model. For example, on a physiological level, ELA leads to a distortion of the ANS not only during baseline but also in reaction to safe stimuli, driven by a blunted PNS activity. This limits the ability to recover from stressful situations, potentially increasing allostatic load and thus increasing the risk of psychopathology^8^. This maladaptation leads to an increase in uncomfortable emotions, which require emotion regulation strategies to be adequately handled. However, ELA has already been linked to poorer emotion regulation skills^44^. This may also be the link between ELA and an increase in health-risk behaviors such as smoking, as these behaviors can be used as maladaptive emotion regulation strategies^44^. This may explain why we found especially emotional abuse to affect the subjective and physiological relaxation response, even in a healthy sample.

Every effect we found is considered small, however, the included sample size was adequate to find a small effect. The sample consisted of relatively young individuals. A previous meta-analysis found the effect of ELA on HRV to be more profound in older samples^2^, highlighting the importance of taking age into account. Additionally, the sample reported overall low ELA scores, which also likely limited the effect of ELA on HRV^2^.

Looking at the employed relaxation interventions, the nature video did not significantly increase physiological relaxation. Possible factors limiting the video’s effectiveness and ideas for its improvement are discussed above.

Future studies should assess different forms of ELA since they seem to have specific influences on HRV and the relaxation reaction. When using the PBI the subscales for both mother and father should be taken into account, as in the present sample only paternal behavior significantly impacted subjective relaxation. Additionally, a broader sample should be assessed, including a wider age range and psychopathology as factors that affect the influence of ELA on HRV. An interesting research question for future studies concerns the relationship between subjective and physiological relaxation parameters, and whether ELA influences this connection. Investigating this could provide insight into how ELA impacts a person’s ability to perceive their own physiological relaxation state. The effectiveness of both relaxation interventions could be improved. For the nature video, the sense of presence could be amplified by increasing the video quality and adding the possibility of interacting with the virtual environment. Preferences for different nature scenes (e.g., forest or beach) could also be considered. For the breathing exercise, breathing rhythm should be measured to check that participants are following the pacer accurately, since in a previous study participants were unable to follow a pacer with six breaths per minute^48^.

In the present study, we found the subjective and physiological relaxation reaction of healthy participants to be blunted in association with ELA, especially emotional abuse. This adds another link to the relationship between ELA and psychopathology, indicating that not only resting state HRV is reduced but also the physiological and subjective relaxation capabilities are blunted, even in a healthy sample. Many effective therapeutic approaches include relaxation techniques (e.g., Progressive Muscle Relaxation^49^) hinting that increasing relaxation capabilities are an important aspect of the treatment of psychopathology. Whether it is possible to reduce the risk for mental and physiological diseases arising from experiencing ELA by regularly performing relaxation exercises is still unclear. Nevertheless, it constitutes a promising interventional approach, as it could be an important aspect in the prevention and treatment of disease, increasing health and wellbeing in the population.

## Methods

### Participants

We recruited participants at the University of Konstanz between December 2022 and May 2023 for a total of 117 participants. Of those five were excluded as they only attended one experimental session, two were excluded due to technical difficulties, and seven were excluded as they indicated suffering from a mental disorder. Thus, the final sample consists of 103 participants (see Table 3). Inclusion criteria were as follows: fluency in German, being mentally and physically healthy (especially no diabetes, epilepsy, heart diseases, or cardiac pacemakers), having a body mass index (BMI) between 18.5 and 29.9, and currently not working night shifts. Participants were asked to refrain from consuming caffeine, alcohol, and nicotine for four hours before the experiment, and intensive sports 12 h before the experiment. Those criteria were implemented to minimize confounding effects on HRV^7,30,31,11,50^ and to minimize the risk for participants since virtual reality glasses can trigger epileptic seizures^51^. Participants received 20 € or course credit for their participation. The study procedure was approved by the Ethics Committee of the University of Konstanz and followed the guidelines of the Declaration of Helsinki.

**Table 3:** Sample characteristics.

	Total sample N = 103
	*M* ± *SD*
Sex	
Female	n = 65 (63.11%)
Male	n = 38 (36.89%)
Age	22.73 ± 3.43
BMI	22.20 ± 2.67
Alcohol consumption	5.55 ± 03.15
Nicotine use	0.52 ± 1.91
Depressive symptoms (BDI)	7.07 ± 5.26
Parental bonding (PBI)	
Without father figure	n = 6
Father overprotection^a^	8.03 ± 6.74
Father care^a^	25.16 ± 6.72
Without mother figure	n = 2
Mother overprotection^b^	11.35 ± 8.26
Mother care^b^	28.93 ± 6.56
Childhood trauma (CTQ)	
Emotional abuse	8.20 ± 3.90
Physical abuse	5.85 ± 2.60
Sexual abuse	5.78 ± 2.53
Emotional neglect	8.57 ± 3.63
Physical neglect	6.60 ± 2.08

Female and male describes the biological sex assigned at birth; BMI = Body Mass Index; Alcohol = glasses of alcoholic beverage consumed per week; Nicotine = cigarettes consumed per day; BDI = Beck Depression Inventory, range: 0–63; PBI overprotection range: 0–39; PBI care range: 0–36; CTQ range per subscale: 5–25.

^a^data collected from n = 97 participants.

^b^data collected from n = 101 participants.

### Study procedure

**Fig. 3:**
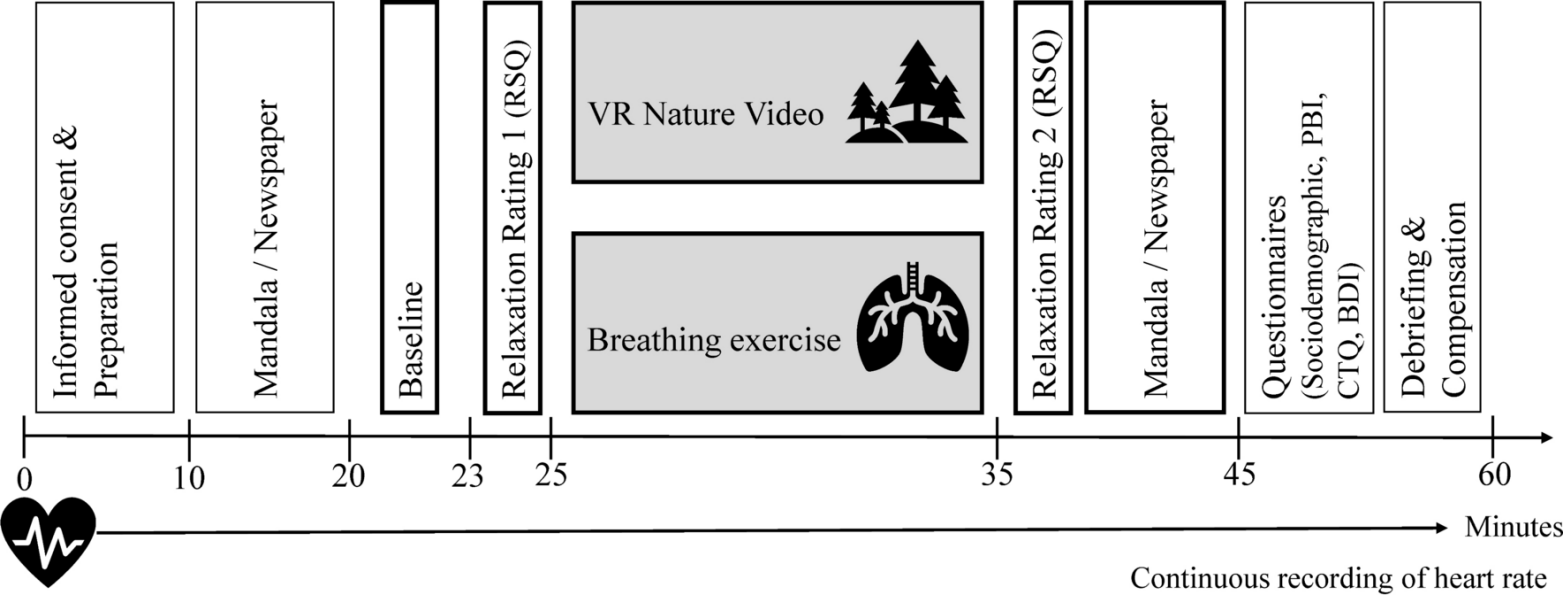
Graphical representation of the within-study design. The bold frames indicate which phases of the experiment were included in the HRV analyses.

Both sessions of the within-design took place five to nine days apart to minimize carry-over effects, with the order of experimental conditions randomized. At the first session participants were informed of the experimental procedure and provided their written informed consent. Subsequently, both sessions followed the same procedure, see Fig. 3 for a graphical representation of the study procedure. For the first ten minutes, participants could choose between a newspaper or mandala to let their heart rate reach a resting baseline. During the baseline period, participants viewed a fixation cross for three minutes. Thereafter participants rated their subjective relaxation using the RSQ. The seven-minute relaxation intervention followed, wherein participants either watched a nature video or completed a paced breathing exercise. Subsequently, they completed the RSQ for the second time. This was followed by a ten-minute interval in which participants were again given the choice between a newspaper and mandala. Finally, various questionnaires were completed (sociodemographic and ELA questionnaires). With this design, we followed the recommendations for HRV assessment^30^.

### Relaxation intervention

**Fig. 4:**
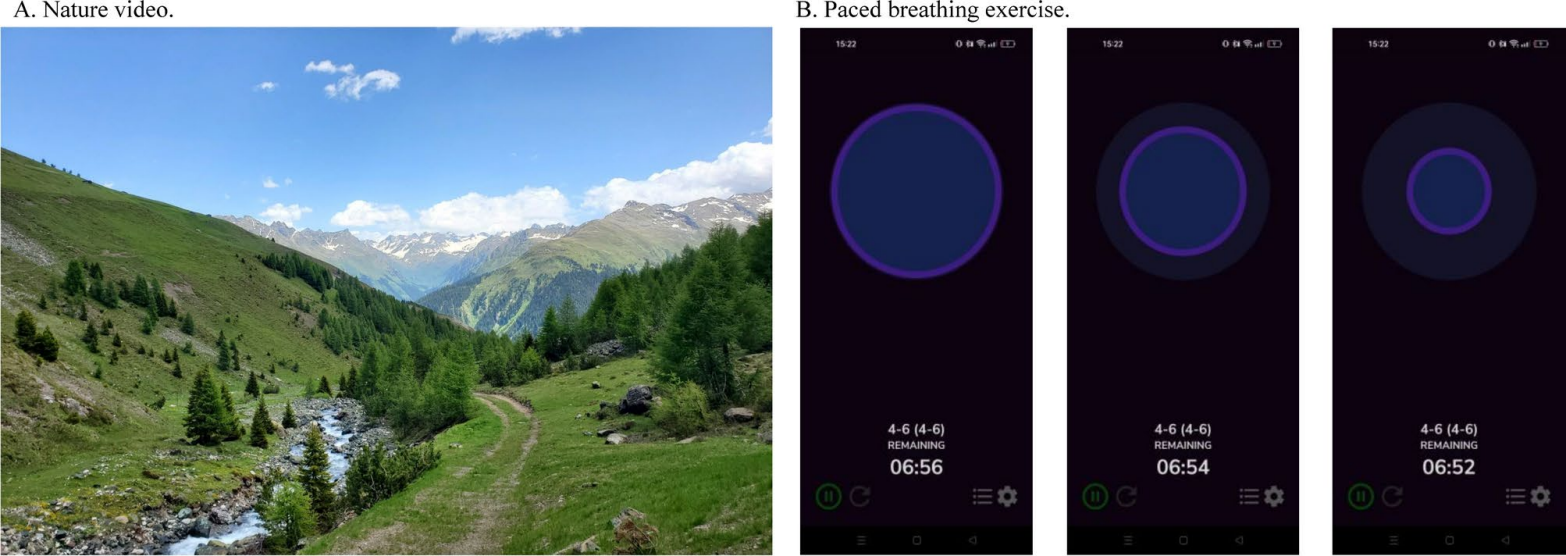
Depictions of both relaxation interventions.

Two relaxation interventions were conducted since the mechanisms behind their relaxing effects are postulated to differ. The nature relaxation intervention (“nature”) was a 360° video depicting mountains, a flowing river, green meadows, and trees with the associated sounds. The video was recorded in the Swiss Alps near Davos using an Insta360 Pro 2 video camera (Insta360, California, USA). Participants watched the video using virtual reality glasses (Meta Quest 2; Meta Platforms Inc., California, USA), giving them the ability to experience the view by turning their heads. For the breathing intervention (“breath”) participants followed a pacer, guiding them to breathe six breaths per minute (four seconds inhale, six seconds exhale). The pacer was presented as an increasing and decreasing circle on an iPad using the app “Awesome Breathing: Pacer Timer”^52^, see Fig. 4.

### Measurements

*Physiological relaxation measurement* To measure relaxation on a physiological level participants applied the Polar H 10 sensor (Polar Electro GmbH Deutschland, Germany) to their chest, which was connected via Bluetooth to the “HRV Logger” app^53^ running on iPads.

*Subjective relaxation measurement* To measure subjective relaxation, the RSQ^28^ was used to measure relaxation at the current moment directly before and after the intervention. To calculate the total RSQ score all ten items are added with higher values indicating a higher level of relaxation. The RSQ allows to derive changes induced by a short-term relaxation intervention with reliability rated as high (ω = 0.83^28^). In our sample, Cronbach’s *α* ranged from 0.60 for the first RSQ assessment to *α* = 0.71 after the intervention.

*Early life adversity* We employed two questionnaires assessing ELA retrospectively. The PBI (original:^54^; German translation:^35^) assesses the parenting style of mother and father. For each parent, the items are cumulated in the subscales “care” (12 items) and “overprotection” (13 items). A low care score and/or a high overprotection score are considered forms of ELA. For the German translation, reliability is considered high

(Cronbach’s α = 0.86–0.95^35^, current sample: paternal care α = 0.90, paternal overprotection α = 0.89, maternal care α = 0.91, maternal overprotection α = 0.93). The CTQ (original^55^; German translation^35^) assesses five different forms of abuse and neglect with five items each: emotional abuse, physical abuse, sexual abuse, emotional neglect, and physical neglect. For each subscale, the items are added up, with higher values indicating a higher level of abuse or neglect. It is a widely used questionnaire to assess ELA retrospectively^34^, with high reliability for the total questionnaire (Cronbach’s α = 0.94^36^). In our sample, we found similar values of Cronbach ‘s α in all subscales (sexual abuse α = 0.95, physical abuse α = 0.90, emotional abuse α = 0.88, emotional neglect = 0.86) except for physical neglect (α = 0.13). A possible explanation for the lower Cronbach’s α value observed, could be the relatively low physical neglect rates within our sample, as well as the smaller sample size compared to those typically used for questionnaire validation.

*Covariates* Various covariates influence HRV^7,30^. We chose to assess and include BMI, age, sex, alcohol consumption (glasses of alcoholic beverages per week), nicotine consumption (cigarettes per day), and depressive symptoms measured with Becks Depression Inventory (BDI-II; original:^56^; German translation:^57^) as covariates.

### Data preprocessing and analyses

R^58^ with the R Studio interface^59^ was used to preprocess the heart rate data to minimize the influence of artifacts, outliers, and missing entries. In case of outliers, no more than 3% of data was changed, missing entries were interpolated as long as no more than ten seconds of consecutive entries were missing, and artifacts were removed. Using the package RHRV^60^ the HRV marker RMSSD was calculated for three-minute intervals (one interval at baseline, two intervals during the relaxation intervention, and three intervals subsequently). We also assessed high-frequency HRV, however as it only represents vagal tone adequately within breathing rates of nine to 24 cycles per minute^61^, we chose to focus on RMSSD. To minimize the effects of statistical outliers for all dependent variables we employed winsorizing (± 3*SD*).

For subjective relaxation, we conducted a mixed ANOVA to analyze the effects of both relaxation interventions as predictors on changes in total RSQ scores from pre to post intervention as outcome. To test the influence of ELA on the subjective relaxation response we conducted mixed ANOVAs with each PBI subscale and CTQ subscale as predictors in addition to type of intervention as predictor and changes in total RSQ scores as outcome variable.

For physiological relaxation we employed multilevel models (MLM) for changes in RMSSD induced by both interventions since the data are nested (repeated RMSSD measures nested within participants nested within conditions) and the differences between participants (random effects) are greater for physiological measures than for questionnaire data. There were no missing data. To estimate the model maximum likelihood was used.

Graphs were created using the package ggpubr^62^, for calculating ANOVAs and ANCOVAs we used the package ez^63^, and for multilevel models the package multilevel^64^.

## Data Availability

The dataset collected and analysed during the current study is available from the corresponding author on reasonable request.
